# Impact of Patient Characteristics on the Outcomes of Patients with Gastrointestinal Cancers Treated with Immune Checkpoint Inhibitors

**DOI:** 10.3390/curroncol30010060

**Published:** 2023-01-06

**Authors:** Hyejee Ohm, Omar Abdel-Rahman

**Affiliations:** 1 Department of Medicine, University of Alberta, Edmonton, AB T6G 1Z2, Canada; 2 Department of Oncology, Cross Cancer Institute, University of Alberta, Edmonton, AB T6G 1Z2, Canada

**Keywords:** GI cancer, digestive malignancies, ICIs, immunotherapy

## Abstract

Gastrointestinal (GI) cancers are a group of malignancies that globally account for a significant portion of cancer incidence and cancer-related death. Survival outcomes for esophageal, gastric, pancreatic, and hepatobiliary cancers remain poor, but new treatment paradigms are emerging with the advent of immune checkpoint inhibitor (ICI) therapy. This review characterizes patient-related prognostic factors that influence the response to ICI therapy. We performed an analysis of the landmark randomized clinical trials in esophageal, gastric, colorectal, hepatocellular, pancreatic, and biliary tract cancers in terms of patient demographic factors. A literature review of smaller retrospective studies investigating patient-related factors was completed. The immunological bases for these associations were further explored. The key predictive factors identified include age, sex, performance status, geography, body mass index, sarcopenia, gut microbiome, various biochemical factors, and disease distribution.

## 1. Introduction

Gastrointestinal (GI) cancers are a heterogeneous group of malignancies that account for a significant portion of cancer-related morbidity and mortality worldwide. In 2018, it comprised 26% (4.8 million) of global cancer incidence and 35% (3.4 million) of all cancer-related deaths [1]. GI cancers can originate anywhere along the gastrointestinal tract, including the esophagus, stomach, colon/rectum, hepatobiliary system, or pancreas. They are distinct in their etiology, pathophysiology, and epidemiologic profile. Apart from colorectal cancer, most have a poor prognosis. The five-year survival rates for esophageal and gastric cancer are in the 20–25% range, while for hepatobiliary and pancreatic cancer, the estimate is closer to 10–15% [1,2,3,4,5].

Immune checkpoint inhibitors (ICIs) have drastically changed the landscape of cancer treatment. In colorectal cancer, monotherapy with PD-1 inhibitors is ineffective in microsatellite stable (MSS) disease. However, it does show promising efficacy when used as a first-line treatment for microsatellite instability-high (MSI-H) tumors, with a more tolerable side effect profile [6]. In gastric cancer, monotherapy with PD-1 inhibitors in the first-line setting showed no benefit compared to standard chemotherapy, but a combination treatment with nivolumab and chemotherapy resulted in a significant improvement in overall survival (OS) and progression-free survival (PFS) compared to chemotherapy alone [7,8].

Several factors can impact the outcomes of GI cancer patients treated with ICIs. Although the research focus has been on molecular factors (e.g., PD-L1 expression; MSI status; or tumor mutational burden), it is undeniable that patient-related factors (e.g., age, sex, and performance status) can modulate the impact of ICIs on cancer patients in general and GI cancer patients in particular. This review aims to identify the various patient characteristics and clinical factors associated with the response to ICIs and explores the immunological basis for these associations. 

### 1.1. Key Immune Cells in Cancer Immunomodulation

**Figure 1:**
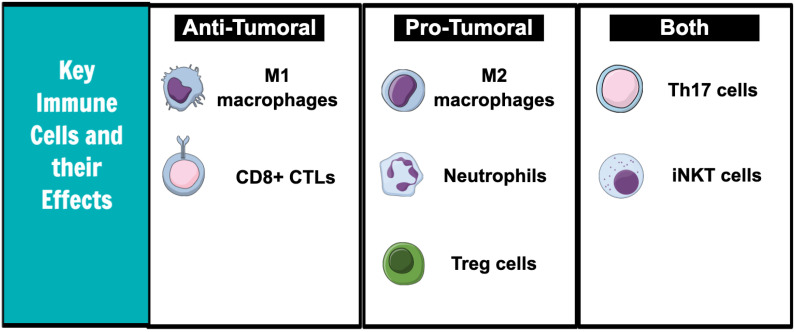
Key immune cells and effects on tumor microenvironment.

The immune system is composed of innate and adaptive responses. Both immune systems are critical for modulating the growth and progression of tumor cells (Figure 1). 

### 1.2. Innate Immune Cells

Neutrophils have pro-tumoral effects. Neutrophils in the tumor microenvironment release vascular endothelial growth factor (VEGF), which increases tumor angiogenesis [9] and produces nitric oxide (NO) and reactive oxygen species (ROS) that promote carcinogenesis by inducing DNA damage [10]. In addition, the cytokines released by neutrophils may enhance tumor cell invasiveness by inducing the expression of specific cell surface receptors. For example, in bronchoalveolar carcinoma, the cytokines released by tumor cells caused the neutrophils to release hepatocyte growth factor (HGF), which then increased tumor cell migration and spread [11]. HGF has been proven to also increase the invasive capacity of hepatocellular carcinoma and cholangiocarcinoma [12].

Macrophages exist in M1 or M2 phenotypic states and have both pro-tumoral and anti-tumoral effects [13]. M1 macrophages are anti-tumoral and produce inflammatory cytokines, such as TNF-a and IL-6 [14]. They are typically stimulated by cytokines released by Th1 cells. Under the influence of IL-4 and IL-13, however, the macrophages polarize to the M2 state. The pro-tumoral M2 macrophages produce high levels of anti-inflammatory IL-10 and have a poor antigen-presenting capacity [15]. Cytokines released by the M2 macrophages recruit anti-inflammatory T regulatory and Th2 helper cells [16]. 

Invariant natural killer T cells (iNKT) are innate-type T cells and have both anti-tumoral and pro-tumoral effects as well. It can suppress cancer progression by either directly binding to the tumor cells and causing cell lysis or indirectly by activating the natural killer (NK) cells of the innate immune system [17]. iNKT cells have mechanisms to activate anti-tumoral T helper cells rapidly [18]. In human models of colorectal cancer, an increased iNKT frequency is linked to an improved prognosis [19]. Contrarily, iNKTs in adipose tissue release IL-10, which maintains macrophages in the pro-tumoral M2 state [20].

### 1.3. Adaptive Immune Cells

CD8+ cytotoxic T cells (CTLs) kill tumor cells. They recognize neoantigens on tumor cells and insert cytotoxic granules into the intracellular environment, causing tumor cell lysis [21]. Evidence of CTL infiltration into the tumor microenvironment has been associated with longer disease-free survival in numerous solid tumors, including colorectal, pancreatic, and hepatocellular cancers [22]. 

Th17 cells have been associated with either pro-tumoral or anti-tumoral responses based on the tumor type. In gastric cancer cell lines, Th17 cell-mediated IL-17 levels have been shown to increase neutrophil recruitment and thus increase gastric cancer progression [23]. An analysis of the tissue samples of 52 patients with colorectal cancer revealed that high IL-17 levels were associated with an increased vascular density and that this process was mediated by an increased VEGF production [24]. However, Th17 cells have also been shown to cooperate with CTLs to induce tumor cell lysis in some murine tumor models [25]. Further characterization of the Th17-mediated pathways is needed to understand the full extent of its effects on cancer.

T regulatory cells promote cancer growth. T regulatory cells are linked to poor outcomes in patients with colorectal and hepatocellular cancers [26,27]. They exert their anti-inflammatory effect by producing immunosuppressive cytokines, such as IL-10 and IL-19, and also by dampening the functions of other immune cells through direct cell contact [28]. For example, in colorectal cancer, the presence of both the T regulatory cells and effector T cells leads to overall immunosuppressive responses in the tumor microenvironment [26]. T regulatory cells have also been found to co-express PD-1 and CTLA-4 receptors, which further dampens the anti-tumor immune response [29].

## 2. Demographic Factors and Outcomes of ICIs

### 2.1. Age

Conflicting reports have been published on the influence of older age on the immune system’s function. Advanced age has routinely been linked with immune senescence in geriatric literature, with progressive sarcopenia being a potential mechanism [30]. Muscle mass and function decline with age, leading to immunological dysregulation [31]. In contrast, older age is associated with higher tumor mutational burden, which may improve the response to ICI therapy. The tumor mutational burden in patients with hepatocellular and gastric cancer patients aged ≥ 60 and <60 was found to be different, with a significant positive correlation between the two variables [32]. 

A thorough review of unstratified subgroup analyses from landmark GI cancer randomized clinical trials (RCTs) suggests that age is most often not associated with significant changes in ICI benefits (Table 1). However, in two RCTs, younger patients tended to fare better. In Checkmate-577, comparing the outcomes of adjuvant nivolumab in resected esophageal and gastroesophageal junction cancer patients, who were previously treated with neoadjuvant chemoradiation, patients aged < 65 derived statistically significant benefits from nivolumab (HR 0.65, 95% CI [0.51–0.84]), whereas patients aged ≥ 65 did not (HR 0.80, 95% CI [0.57–1.12]) [33]. Similarly, in the landmark trial by André et al., investigating single-agent pembrolizumab compared to chemotherapy in patients with metastatic MSI-H colorectal cancer, patients aged < 70 saw an OS benefit from pembrolizumab (HR 0.52, 95% CI [0.37–0.75]), whereas those aged ≥ 70 did not (HR 0.77, 95% CI [0.46–1.27]) [6]. It should be noted that these observed age-related differences might be related to tolerability factors (i.e., older patients might have less treatment tolerability and higher treatment-related complications/ interruptions) rather than specific immunological mechanisms.

### 2.2. Sex

Women have stronger immune responses compared to men. Though males demonstrate stronger innate immune responses, the adaptive immune system is much more potent in women. Women mount stronger responses to vaccinations, are more likely to develop autoimmune disease, and have 40% less viral RNA in their blood than men during acute HIV infection [39]. In in vitro settings, women mount a higher number of activated T helper cells and CTLs in the peripheral blood compared to men following stimulation of the peripheral blood mononuclear cells [40]. The upregulation of more antiviral genes and pro-inflammatory genes leads to greater functionality of female CTLs compared to male CTLs [41].

Despite this, being female has been linked to a poorer response to immunotherapy. One of the main hypotheses is that tumor cells in women have developed stronger mechanisms for immune escape due to their stronger immune surveillance systems [42]. Additionally, as the risk of autoimmune diseases is higher in women, treatment may be aborted earlier due to the side effects [42]. Finally, male pancreatic, colorectal, gastroesophageal, hepatocellular, and biliary tract tumors exhibit higher tumor mutational burdens when compared to females [43].

The subpar responses to immunotherapy in females compared to males are also seen in several GI cancer RCTs (Table 2). KEYNOTE-590 evaluated pembrolizumab plus chemotherapy compared to chemotherapy alone in patients with unresectable esophageal cancer. The subgroup analyses revealed that there was no statistically significant benefit seen in females (HR 0.89, 95% CI [0.59–1.35]), whereas males did see an OS benefit with combination pembrolizumab and chemotherapy (HR 0.70, 95% CI [0.58–0.84]) [34]. Similarly, inferior responses to adjuvant nivolumab in female esophageal cancer patients were seen in the Checkmate-577 trial [33]. In patients with unresectable gastric cancer, treatment with nivolumab plus chemotherapy showed no significant improvements in the OS for females (HR 0.78, 95% CI [0.59–1.03]) in contrast to males (HR 0.67, 95% CI [0.56–0.80]) [8]. These findings are corroborated in the HIMALAYA trial, which found that tremelimumab and durvalumab did not lead to a statistically longer OS in female unresectable HCC patients (HR 1.02, 95% CI [0.67–1.56]), but did have an effect on male patients (HR 0.73, 95% CI [0.61–0.88]) [37].

### 2.3. Performance Status

Performance status, as evaluated by the Eastern Cooperative Oncology Group (ECOG) scale, among others, has been linked to the variable outcomes of ICI-based treatments. The exact mechanisms driving these differences are unknown, however, as there is a scarcity of research investigating the relationship between performance status and the immune system.

Most RCTs limit the patient’s eligibility to physiologically fit individuals with an ECOG status of 0 or 1. The differences in response to ICI therapy between these two groups are overall inconsistent (Table 3). The Checkmate-649 trial, evaluating a combination of nivolumab and chemotherapy in gastric cancer patients, revealed superior outcomes with ICI therapy in those with ECOG 1 (HR 0.63, 95% CI [0.52–0.76]) but not among those with ECOG 0 (HR 0.79, 95% CI [0.61–1.02]) [8]. Similarly, improved outcomes with ICI were seen with ECOG 1 but not with ECOG 0 patients within the IMbrave-150 study evaluating atezolizumab–bevacizumab in unresectable HCC patients and the TOPAZ-1 study evaluating durvalumab plus chemotherapy in patients with unresectable biliary tract cancer [36,38]. In contrast, André et al. found that patients with ECOG 0 derived more benefit from pembrolizumab monotherapy (HR 0.37, 95% CI [0.24–0.59]) compared to those with ECOG 1 MSI-H colorectal cancer patients (HR 0.84, 95% CI [0.57–1.24]) [6].

Despite the inconsistent findings, three landmark GI RCTs suggested a superior outcome with ICI therapy in ECOG 1 vs. ECOG 0 patients. This seemingly contradictory finding may be explained by the baseline healthy nature of ECOG 0 patients. They derive benefits from both chemotherapy as well as ICI with limited side effects; thus, the differences in outcomes between ICI and chemotherapy may be less apparent. We must also note that the above discussion does not account for the impact of an ECOG performance score > 1 on the outcomes of ICIs, as this was not included in the evaluated RCTs. In a smaller retrospective cohort study of esophageal cancer patients treated with nivolumab, ECOG ≥ 2 was associated with a reduced OS (HR 17.9, 95% CI [4.96–64.7]) compared to patients with ECOG ≤ 1 [44].

### 2.4. Geography

Significant associations between geographic regions and the response to ICIs are seen in GI cancers (Table 4). Both HCC and biliary tract cancers show superior effects of ICI in Asian regions. In unresectable HCCs, the COSMIC-312 trial evaluating atezolizumab and cabozantinib demonstrated statistically significant benefits for the OS in Asian regions (HR 0.56, 95% CI [0.32–0.87]) but not in non-Asian regions (HR 0.74, 95% CI [0.54–1.02]) [45]. Similar associations were seen in the HIMALAYA trial [37]. The TOPAZ-1 study showed the addition of durvalumab to standard chemotherapy in biliary tract cancers improved the OS in Asian regions (HR 0.72, 95% CI [0.56–0.94]) but not in other regions (HR 0.89, 95% CI [0.66–1.19]) [38]. Differing etiologies of malignancy likely underlie this finding. HCC in Asian countries is often secondary to chronic infections with hepatitis B or hepatitis C, whereas, in non-Asian countries, it is partly driven by alcohol or NASH [1]. Hepatic inflammation and cirrhosis are closely linked to the development of biliary tract cancers as well [46].

NASH-related HCC demonstrates dysfunctional tumor immune surveillance mechanisms in contrast to HCC driven by other causes. In mice fed with a high-fat diet who developed NASH and eventually HCC, there was an increased number of PD1+ CTLs in the liver, but these cells demonstrated features of exhaustion and were unable to exert their effector functions [47]. Exhausted CTLs demonstrate increased inhibitory receptors, decreased cytokine production, and depressed cytolytic functions [48]. In mice with NASH-HCC, treatment with the anti-PD1 agents did not lead to tumor regression but led to increased fibrosis, whereas the non-NASH mouse models of liver cancer reacted to PD-1 therapy appropriately with tumor regression [47].

Similarly, in gastric cancer, Checkmate-649 demonstrated a significant benefit to the addition of nivolumab to standard chemotherapy in Asian regions (HR 0.64, 95% CI [0.47–0.87]) but not in Canada or the USA (HR 0.67, 95% CI [0.43–1.03]) [8]. Again, the difference is likely attributable to the etiology. In low human development index settings, which include certain parts of Asia, most gastric cancers are of non-cardia origin and secondary to chronic H. pylori infection. In contrast, in high human development index settings, gastric cancers are more likely of a cardia origin secondary to obesity [1].

## 3. Body Composition

### 3.1. Body Mass Index

Human white adipose tissue is known to function as an immune organ [18]. With normal weight, adipose tissue has an anti-inflammatory predilection and is composed of a combination of iNKT cells, T regulatory cells, and M2 macrophages [18]. Obesity tips the scales toward a pro-inflammatory state. In obese mouse models and human patients, the overall numbers of T regulatory cells are lower compared to lean mice [49]. M2 macrophages are polarized to the M1 state and produce substantial amounts of pro-inflammatory cytokines, such as IL-6 and TNF-α [50]. As adipose tissue grows in obesity, the iNKT cells become depleted [20]. Loss of the iNKT cells decreases the production of IL-10, which is crucial in maintaining macrophages in the M2 state.

Apart from a change in immune composition, obesity also increases the overall count of immune cells in the human body. Ilavska et al. found a significant difference in the number of leukocytes in normal weight (6.3 × 10^9^ cells/μL) compared to obese (7.3 × 10^9^ cells/μL) and extremely obese (8.4 × 10^9^ cells/μL, *p* < 0.001) individuals [51]. The BMI was correlated with increased numbers for almost all the leukocyte subpopulations, including neutrophils, macrophages, and lymphocytes.

Due to the pro-inflammatory effect of obesity, being overweight has been linked to favorable outcomes in various solid tumors. Among patients with advanced hepatocellular carcinoma, the median OS in patients treated with anti-PD1 antibody was significantly higher in patients with a body mass index (BMI) of ≥25 compared to those with a BMI of <25 (17.5 vs. 5 months, *p* = 0.0034) [52]. Similar findings were reported in a large multicentre retrospective cohort study conducted by Cortellini et al., comprising patients with various solid tumors treated with ICIs. Patients with a BMI of ≥25 had improved objective response rates (41.3% vs. 20.9%, *p* < 0.0001), a median PFS (11.7 vs. 3.7 months, HR 0.46, 95% CI [0.39–0.54]), and a median OS (26.6 vs. 6.6 months, HR 0.33, 95% CI [0.28–0.41]) compared to those with a BMI of <25 [53]. Other measures of adipose tissue, including the visceral adipose tissue index based on baseline CT imaging, have shown similarly positive associations in GI cancer patients [54].

### 3.2. Sarcopenia

Skeletal muscles have emerged as endocrine organs capable of producing myokines (i.e., cytokines released from the muscles), such as IL-15 and IL-6. IL-15 protects the NK cells from apoptosis, stimulates CTL homeostasis, enhances neutrophil recruitment, and promotes the survival of naïve T and B cells [55,56]. The pulsatile release of cytokines by skeletal muscles in normal conditions of physiologic stress, such as exercise, is crucial for skeletal muscle hypertrophy and anabolism [30].

The reduced muscle mass seen in sarcopenia is primarily mediated by chronic, low-grade inflammation. In contrast to pulsatile exposure, prolonged exposure to IL-6 has been shown to facilitate muscle atrophy by blunting muscle anabolism [57]. Reduced skeletal muscle mass, in turn, leads to reduced myokine production in response to new immune insults. Furthermore, chronic low-grade inflammation leads to CTL exhaustion. Clinically, the presence of sarcopenia predicts the risk of infection after surgery, nosocomial infections after three weeks of hospitalization, and community-acquired pneumonia in the outpatient setting [58,59,60]. Taken together, sarcopenia is associated with an immunosuppressed state.

Sarcopenia has been repeatedly associated with poor OS in cancer patients [52,54,61,62], which extends to ICI treatment outcomes. Cachexic gastric cancer patients previously treated with chemotherapy who received nivolumab had a significantly worse median OS (2.3 vs. 6.6 months, HR 2.65, 95% CI [1.28–5.49]) and time to treatment failure (1.8 vs. 2.6 months, HR 2.65, 95% CI [1.28–5.49]) compared to non-cachectic patients [61]. Among patients with primary liver cancer treated with ICIs, a low skeletal muscle index, based on CT imaging one month before the treatment started, was associated with a poor OS (HR 5.39, 95% CI [1.74–16.74]).

### 3.3. Gut Microbiome

The gut microbiome has emerged as one of the more mysterious immune regulators in recent years. The exact interplay between microbiota and the immune system is beyond the scope of this paper, but briefly, bacteria in the gut interact with the stromal and epithelial cells to regulate barrier function, prevent pathogen infection, and control the overgrowth of pathological organisms [63]. The microbiome is also the source of various toxic metabolites and carcinogenic products that can directly promote tumor growth [64]. In animal models, a response to ICI therapy was correlated with a gut microbiome composition. Mice showing a beneficial response to the PD-1 therapy had an increase in the *Bifidobacterium* species, and by co-housing the non-responding mice with those with a good response, the beneficial effect was transferred [65].

A response to ICI therapy was found to differ according to the gut microbiome composition in humans as well. In patients with advanced hepatobiliary cancers treated with ICIs, a higher abundance of *Lachnospiraceae bacterium-GAM79* achieved a longer PFS (7.9 vs. 13.8 months, *p* = 0.020) and OS (not reached vs. 13.8 months, *p* = 0.023) [66]. In contrast, a *Veillonellaceae* species abundance was associated with a decreased PFS (4.2 vs. 6.9 months, *p* = 0.018) and OS (8.9 vs. 22.3 months, *p* = 0.001) [66].

A more clinically useful association is the one observed between recent antibiotic use and ICI treatment response [67,68]. Antibiotics eradicate microorganisms in the gut and alter their baseline composition. Greally et al. found that in esophagogastric cancer patients, treatment with antibiotics 30 days before ICI was associated with a worse OS (0.9 vs. 5.8 months, HR 2.40, 95% CI [1.30–4.20]) with no significant difference in PFS. Interestingly, this adverse association disappeared when the timing between antibiotic therapy and ICI treatment was increased to 60 days [67].

## 4. Biochemical Factors

### 4.1. Neutrophil to Lymphocyte Ratio

As described previously, the presence of neutrophils in the tumor microenvironment is associated with pro-tumoral activity due to their tendency to stimulate angiogenesis, cause DNA damage via NO and ROS production, and release cytokines that promote tumor invasiveness [9,10,11]. In contrast, the presence of lymphocytes, especially CTLs, has been associated with anti-tumoral effects. The ratio of neutrophils to lymphocytes (NLR), which is very easily calculated based on the baseline laboratory markers, is useful in predicting a prognosis among patients treated with ICIs in many different solid tumors.

A retrospective cohort study of hepatocellular carcinoma patients by Akce et al. showed that the neutrophil to lymphocyte ratio, when calculated two weeks prior to immunotherapy initiation, was found to be predictive of the treatment outcomes. Patients with a baseline NLR of ≥5.15 had a shorter median OS at 3.6 months compared to those with an NLR of <5.15, who had a median OS of 14.3 months (*p* < 0.001) [52]. Other studies suggest that it is the degree of change in the NLR following ICIs that has a prognostic value. For example, among 60 patients with metastatic esophageal squamous cell carcinoma treated with an anti-PD-1 agent, a ≥1.4-fold increase in the NLR from baseline after one cycle was associated with significantly unfavorable outcomes in both PFS (1.4 vs. 2.1 months, HR 2.68, 95% CI [1.18–6.09]) and OS (1.8 vs. 8.9 months, HR 3.19, 95% CI [1.46–6.97]) [69]. Similarly, among patients with gastric cancer, there were significant differences in the NLR four weeks after ICI treatment in those with complete and partial responses (*p* = 0.044) [70].

### 4.2. C-Reactive Protein

CRP is widely used in clinical medicine as a non-specific inflammatory marker. Some in vitro studies suggest that CRP, as a molecule, exerts immunogenic effects, but the literature is ambiguous as to whether it is pro-tumoral or anti-tumoral. In order to determine the effect of CRP on the macrophage phenotypic differentiation, monocytes incubated with CRP at concentrations of 0–50 μg/mL were allowed to differentiate into macrophages for 7 days. The characterization of macrophages with flow cytometry revealed that the CRP treatment resulted in an increased population of M1 macrophages. These findings were further corroborated in vivo by administering human CRP to rats [71]. On the contrary, an immunohistochemical measurement of clear-cell renal cell carcinoma tissue sections revealed that patients with high CRP levels had higher levels of T regulatory cells in the tumor microenvironment [72]. CRP has also been shown to downregulate tumor necrosis-related apoptosis-inducing ligand (TRAIL), which induces apoptosis in cancer cells via death receptors. Exposing monocytes to recombinant CRP in an in vitro setting for 24 h was associated with a dose-dependent decrease in TRAIL mRNA levels (*p* < 0.005) [73].

Various scores incorporating the baseline CRP levels have been found to have prognostic value in GI cancers. The Glasgow Prognostic Score (GPS) is a predictive marker incorporating elevated CRP and low albumin. Patients receive a score of 0, 1, or 2 based on the number of abnormalities present. In patients with esophageal squamous cell carcinoma treated with nivolumab or pembrolizumab, having a GPS of ≥1 was associated with a lower PFS (1.6 vs. 4.5 months, HR 2.35, 95% CI [1.19–4.64]) and median OS (4.3 vs. 10.1 months, HR 2.93, 95% CI [1.40–6.10]) [68]. Similar results have been reported in gastric cancer patients [74,75]. In hepatocellular carcinoma, patients with 0, 1, or 2 CRAFITY scores based on AFP (≥100 ng/mL) and CRP (≥10 mg/L) had variable OS (27.6 vs. 11.3 vs. 6.4 months, *p* < 0.001) and disease control rates (80% vs. 68% vs. 46%, *p* < 0.001) following immunotherapy [76].

### 4.3. Serum Sodium Level

Salt exerts an indirect effect on the immune system. High sodium levels promote the development of pro-inflammatory macrophages and lymphocytes. M1 macrophages secrete NO and TNF-α in hyperosmolar environments, and high salt levels have been shown to depress the activity of M2 macrophages [77,78]. Salt also depresses the functions of T regulatory cells by several mechanisms. First, it induces Th17 cells to produce IL-17, which indirectly impairs the functions of the T regulatory cells [48]. Second, it impairs the ability of the T regulatory cells to suppress Th17 cell activity [79]. Third, it transforms the T regulatory cells into a pro-inflammatory phenotype that secretes cytokines such as IFN-γ rather than the classic regulatory IL-10 [79]. Fourth, it skews the differentiation of naïve T cells into the Th17 rather than T regulatory phenotype [80]. Taken together, hyponatremic states would suggest a depressed immune system.

In a sub-analysis from the ATTRACTION-2 clinical trial testing the efficacy of nivolumab in patients with advanced gastric cancer, low sodium levels combined with both an age of <60 and the presence of peritoneal metastasis were found to be associated with a lower benefit from nivolumab therapy [81]. Kim et al. reported on the prognostic effect of hyponatremia among esophageal squamous cell cancer patients as well. Patients treated with either nivolumab or pembrolizumab, who had baseline sodium levels of <135 mmol/L were found to have significantly worse overall survival rates (1.4 vs. 7.4 months, HR 3.27, 95% CI [1.03–10.40]) [68].

### 4.4. Serum LDH

High LDH levels are associated with increased tumoral invasion and migration [82]. LDH-associated lactic acid accumulation compromises both innate and adaptive immune responses. High lactate levels recruit myeloid-derived suppressor cells, which in turn depress the actions of both dendritic and NK cells [83]. Lactate also inhibits the ability of NK cells to secrete pro-inflammatory cytokines, such as IFN-γ [84]. The recruitment of both CTLs and T helper cells to the tumor microenvironment is reduced as the lymphocytes sense high lactate through the cell surface receptors [85]. Preferential differentiation of naïve T cells to T regulatory cells occurs under the influence of myeloid-derived suppressor cells, and high lactic acid levels promote the expression of PD-1 on T regulatory cells [83,86].

Despite the non-specific nature of elevated LDH, it is frequently used as a prognostic marker across many different solid and hematological cancers. A systematic review and meta-analysis of 76 studies conducted by Petrelli et al. showed that the serum LDH level was associated with a worse OS (HR 1.7, 95% CI [1.62–1.79]) and PFS (HR 1.75, 95% CI [1.31–2.33]) across all solid tumors, including gastric, colorectal, and biliopancreatic cancers [87]. In addition to a predictive role of LDH prior to the commencement of treatment, a greater degree of LDH elevation following the initiation of ICI therapy has been associated with treatment resistance. Nakazawa et al. measured the serum LDH-to-albumin ratio in advanced gastric cancer patients after two cycles of nivolumab. In patients with disease progression, there were significantly larger increases in LDH (*p* = 0.012) and a decrease in albumin (*p* = 0.035) [88].

## 5. Disease Distribution

### 5.1. Liver Metastasis

The liver is a common site of distant metastases for GI cancers and demonstrates an immunosuppressed milieu. Certain conditions, such as viral infections, organ transplantation, and autoimmune disease, promote immune tolerance in the liver by inducing CTL anergy and promoting T regulatory cell function [89]. The same mechanism was proven to occur in cancer pathophysiology. Through murine models, Yu et al. showed that liver metastases sequester activated CTLs from the systemic circulation and mediate interactions with macrophages within the liver, leading to CTL apoptosis [90]. In human models, comparisons between patients with and without liver metastases confirmed reduced peripheral T-cell numbers, diversity, and function [90]. Furthermore, the tumor microenvironments of liver metastases compared to their primary tumor differ. An analysis of hepatic metastases in colorectal cancer patients revealed minimal CTL infiltration and higher expressions of myeloid-derived suppressor cells compared to their primary tumor [91].

Greally et al. compared the survival outcomes in patients with metastatic esophagogastric cancer treated with ICIs and found that those with liver metastases had a lower PFS (1.4 vs. 2.1 months, HR 1.5, 95% CI [1.1–2.1]) and OS (3.1 vs. 8.3 months, HR 2.11, 95% CI [1.5–3.0]) compared to those without liver metastases [67]. Worse outcomes were also seen among gastric cancer patients with liver metastases receiving nivolumab as ≥ a third-line therapy [74]. A pan-cancer analysis of 1661 cancer patients across 11 different solid tumor types treated with PD-1/PD-L1 therapy confirmed that the presence of liver metastases was associated with a significantly shorter OS (9 vs. 15 months, HR 1.79, *p* < 0.0001). Interestingly, the presence of liver metastases was not associated with a shorter OS when patients were treated with PD-1/PD-L1 therapy in combination with other treatment modalities, such as radiation, chemotherapy, or CTLA-4 inhibitors [92]. More recently, a phase I/II clinical trial investigated the efficacy of novel ICI agents, botensilimab (CTLA-4 antibody) and balstilimab (anti-PD1 antibody), in heavily pretreated MSS colorectal cancer patients. Full cohort analyses revealed an ORR of 24%, but a much higher ORR of 42% was seen in patients with no history of liver metastases [93].

### 5.2. Peritoneal Metastasis

Similar to liver metastases, there is a difference in the tumor microenvironment between peritoneal metastases and the primary tumor. Peritoneal metastases were found to have a higher proportion of NK cells but lower numbers of Th17 cells and CTLs. There was an upregulation of the angiogenesis-related genes, such as VEGF-A, leading to increased peritoneal neovascularization compared to primary colorectal cancer [94]. In gastric cancer, peritoneal metastases had a higher number of macrophages, but they were phenotypically M2 and expressed higher levels of VEGF [95,96].

A poor ICI response in those with peritoneal metastases has been shown clinically by a prospective cohort study of gastric carcinoma patients treated with ICIs conducted by Tanaka et al. In a multivariate analysis, metastases to the peritoneum were associated with worse OS rates (HR 2.51, 95% CI [1.39–4.55]) [97]. Other studies suggest that it is the presence of ascites and not just peritoneal metastases alone that is associated with poor ICI treatment outcomes. Fuca et al. explored the associations of peritoneal metastases with or without ascites among metastatic MSI-H colorectal cancer patients treated with anti-PD1 therapy. Patients were divided into those with no peritoneal involvement or ascites, those with peritoneal involvement without ascites, and those with peritoneal involvement and ascites. Patients with ascites had significantly reduced two-year PFS (30.4%, HR 2.80, 95% CI [1.65–4.75]) and OS rates (29.7%, HR 3.58, 95% CI [2.06–6.22]). No significant differences, however, were observed in patients without ascites, even if they had peritoneal metastases [98].

## 6. Limitations and Future Directions

Notable limitations of this paper are the inclusion of many retrospective studies, which lack the necessary robustness to make final conclusions about the real-life relevance of the variables discussed. Only the demographic variables, which are consistently reported across the RCTs, were discussed, limiting the inclusion of other patient characteristics, such as smoking status, alcohol use, or ethnicity. Lastly, this review comments on the trends observed in the RCTs, but in the absence of meta-analyses, we cannot make firm conclusions on the significance of these variables across studies.

In the future, patient clinical characterization for ICI therapy may not be limited to demographic or laboratory parameters alone. There is considerable interest in the influence of other tumor microenvironment components, such as the extracellular matrix, in ICI efficacy. For example, elevated type-VIII collagen fragments are seen in patients with colorectal cancer compared to normal controls, and elevated matrix metalloproteinase-9 levels are associated with relapse and poor prognosis in colorectal cancer [35,99]. These molecular markers are exciting avenues for further determining a patient’s suitability for ICI therapy.

## 7. Conclusions

**Figure 2:**
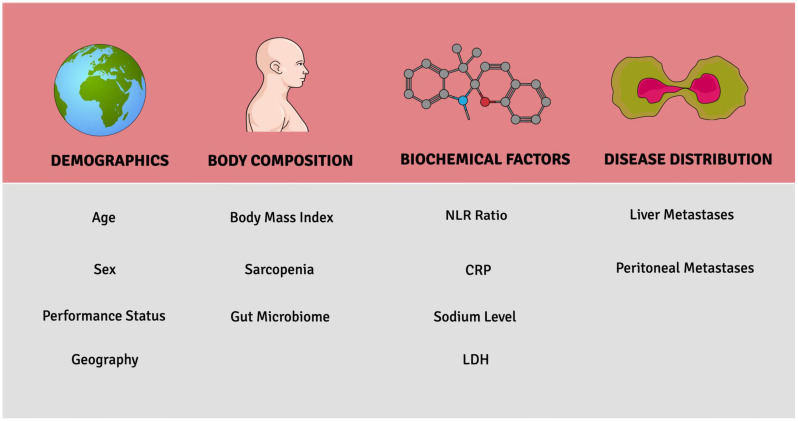
Key patient-related prognostic factors associated with ICI response.

The foray of ICI therapy in GI cancers has led to multiple practice-changing discoveries in the last few years. To date, research on different molecular factors that predict responses to ICI therapy has dominated the literature. In this review, patient-related factors that can modulate the impact of ICIs on GI cancer are explored (Figure 2). Analysis of the landmark RCTs reveals age, sex, performance status, and geography as key demographic factors that might influence the ICI response. Other smaller retrospective studies reveal the clinical relevance of body composition, biochemical factors, and disease distribution. The prognostic factors discussed in this paper may be helpful in clinical settings when determining which patients may benefit from ICI therapy.

## Figures and Tables

**Table 1:** Unstratified hazard ratios for overall survival based on age in landmark GI immunotherapy clinical trials.

Cancer Type	Study Name	Authors/Year	Age	Hazard Ratio	Confidence Interval
Esophageal	KEYNOTE-590	Sun et al., 2021 [34]	<65	0.76	0.61–0.95
			≥65	0.69	0.53–0.89
	Checkmate-577	Kelly et al., 2021 [33]	<65	0.65	0.51–0.84
			≥65	0.80	0.57–1.12
	Checkmate-648	Doki et al., 2022 [35]	<65	0.68	0.47–0.97
			≥65	0.40	0.27–0.61
Gastric	Checkmate-649	Janjigian et al., 2021 [8]	<65	0.69	0.56–0.84
			≥65	0.72	0.57–0.91
Hepatocellular	IMbrave150	Finn et al., 2020 [36]	All patients	0.60	0.44–0.82
			≥65	0.58	0.36–0.92
	HIMALAYA	Abou-Alfa et al., 2022 [37]	<65	0.82	0.65–1.04
			≥65	0.73	0.58–0.93
Colorectal	KEYNOTE-177	André et al., 2020 [6]	<70	0.52	0.37–0.75
			≥70	0.77	0.46–1.27
Biliary Tract	TOPAZ-1	Oh et al., 2022 [38]	<65	0.80	0.61–1.04
			≥65	0.79	0.61–1.04

**Table 2:** Unstratified hazard ratios for overall survival based on sex in landmark GI immunotherapy clinical trials.

Cancer Type	Study Name	Authors/Year	Sex	Hazard Ratio	Confidence Interval
Esophageal	KEYNOTE-590	Sun et al., 2021 [34]	Female	0.89	0.59–1.35
			Male	0.70	0.58–0.84
	Checkmate-577	Kelly et al., 2021 [33]	Female	0.59	0.35–1.00
			Male	0.73	0.59–0.91
	Checkmate-648	Doki et al., 2022 [35]	Female	0.49	0.25–0.97
			Male	0.55	0.41–0.74
Gastric	Checkmate-649	Janjigian et al., 2021 [8]	Female	0.78	0.59–1.03
			Male	0.67	0.56–0.80
Hepatocellular	IMbrave150	Finn et al., 2020 [36]	Female	0.35	0.15–0.81
			Male	0.66	0.47–0.92
	HIMALAYA	Abou-Alfa et al., 2022 [37]	Female	1.02	0.67–1.56
			Male	0.73	0.61–0.88
Colorectal	KEYNOTE-177	André et al., 2020 [6]	Female	0.58	0.39–0.87
			Male	0.59	0.38–0.90
Biliary Tract	TOPAZ-1	Oh et al., 2022 [38]	Female	0.82	0.62–1.08
			Male	0.78	0.60–1.01

**Table 3:** Unstratified hazard ratios for overall survival based on performance status in landmark GI immunotherapy clinical trials.

Cancer Type	Study Name	Authors/Year	ECOG	Hazard Ratio	Confidence Interval
Esophageal	KEYNOTE-590	Sun et al., 2021 [34]	0	0.72	0.55–0.94
			1	0.73	0.59–0.90
	Checkmate-577	Kelly et al., 2021 [33]	0	0.73	0.56–0.96
			1	0.66	0.48–0.89
	Checkmate-648	Doki et al., 2022 [35]	0	0.47	0.31–0.73
			1	0.61	0.43–0.86
Gastric	Checkmate-649	Janjigian et al., 2021 [8]	0	0.79	0.61–1.02
			1	0.63	0.52–0.76
Hepatocellular	IMbrave-150	Finn et al., 2020 [36]	0	0.67	0.43–1.06
			1	0.51	0.33–0.80
	HIMALAYA	Abou-Alfa et al., 2022 [37]	0	0.79	0.63–0.98
			1	0.74	0.57–0.95
Colorectal	KEYNOTE-177	André et al., 2020 [6]	0	0.37	0.24–0.59
			1	0.84	0.57–1.24
Biliary Tract	TOPAZ-1	Oh et al., 2022 [38]	0	0.90	0.68–1.20
			1	0.72	0.56–0.94

**Table 4:** Unstratified hazard ratios for overall survival based on geographic location in landmark GI immunotherapy clinical trials.

Cancer Type	Study Name	Authors/Year	Geographic Region	Hazard Ratio	Confidence Interval
Esophageal	KEYNOTE-590	Sun et al., 2021 [34]	Asian	0.64	0.51–0.81
			Non-Asian	0.83	0.66–1.05
	Checkmate-577	Kelly et al., 2021 [33]	Asian	0.78	0.43–1.41
			Non-Asian	0.69	0.56–0.86
	Checkmate-648	Doki et al., 2022 [35]	Asian	0.57	0.41–0.79
			Non-Asian	0.48	0.29–0.79
Gastric	Checkmate-649	Janjigian et al., 2021 [8]	Asian	0.64	0.47–0.87
			Canada/USA	0.67	0.43–1.03
			Other	0.74	0.61–0.89
Hepatocellular	IMbrave150	Finn et al., 2020 [36]	Asian	0.53	0.32–0.87
			Non-Asian	0.65	0.44–0.98
	HIMALAYA	Abou-Alfa et al., 2022 [37]	Asian	0.71	0.54–0.92
			Non-Asian	0.82	0.66–1.02
	COSMIC-312	Kelley et al., 2022 [45]	Asian	0.56	0.34–0.92
			Non-Asian	0.74	0.54–1.02
Colorectal	KEYNOTE-177	André et al., 2020 [6]	Asian	0.65	0.30–1.41
			Europe/North America	0.62	0.44–0.87
			Other	0.40	0.16–0.98
Biliary Tract	TOPAZ-1	Oh et al., 2022 [38]	Asian	0.72	0.56–0.94
			Non-Asian	0.89	0.66–1.19
